# Towards inhibitors of glycosyltransferases: A novel approach to the synthesis of 3-acetamido-3-deoxy-D-psicofuranose derivatives

**DOI:** 10.3762/bjoc.11.170

**Published:** 2015-09-04

**Authors:** Maroš Bella, Miroslav Koóš, Chun-Hung Lin

**Affiliations:** 1 Institute of Chemistry, Slovak Academy of Sciences, Dúbravská cesta 9, SK-845 38, Bratislava, Slovakia; 2 Institute of Biological Chemistry, Academia Sinica, No. 128 Academia Road Sec. 2, Taipei 11529, Taiwan

**Keywords:** glycosyltransferases, inhibitors, D-psicofuranose, synthesis, thioglycosylation

## Abstract

A novel synthetic strategy leading to 3-acetamido-3-deoxy-D-psicofuranose **9** is presented. The latter compound, after some manipulations, was transformed into fully protected 3-acetamido-3-deoxy-D-psicofuranose **11** as a potential substrate for the synthesis of *N*-acetylglucosaminyltransferase inhibitors designed by computational methods. After the attempted thioglycosylation of **11** with EtSH in the presence of BF_3_·OEt_2_, 2-methyloxazoline derivatives **13** and **14** were isolated.

## Introduction

Glycosyltransferases (GTs) belong to a family of enzymes that are responsible for the biosynthesis of complex oligosaccharides, glycoproteins and other glycoconjugates in mammalian biological systems. These glycoconjugates are operating in the cell and on the cell surface, particularly as glycoproteins, and are involved in many vital biological processes, such as cell–cell communication, signal transduction, activation and response of the immune system etc. [1–2]. On the other hand, an uncontrolled glycosylation caused by genetic mutations of GTs leads to structural changes in various glycoconjugates which contribute to many mammalian diseases [3–4]. In addition, the role played by the glycoconjugates changes markedly during disease development such as malignant transformation [5], cancer cell proliferation and metastases spreading [6]. Since glycosyltransferases (GTs) are entailed in the biosynthesis of glycans and glycoconjugates, which are involved in these disease processes, inhibitors of GTs are of great therapeutic potential and attract remarkable interest for drug development.

Although the mechanism of reactions catalyzed by glycosyltransferases has been investigated thoroughly, many aspects of the catalytic mechanism remain unknown [7]. In general, GTs transfer sugar nucleotide donors onto suitable acceptors during the biosynthesis of glycans and glycoconjugates [8]. Both donor and acceptor substrates are recognized by GTs binding pockets. For instance, in the course of biosynthesis of complex and hybrid oligosaccharides, the insertion of an *N*-acetylglucosaminyl moiety (GlcNAc-) into an oligosaccharide chain was identified as the crucial step catalyzed by *N*-acetylglucosaminyltransferases (GnTs) in the presence of a metal co-factor. In this catalytic reaction, UDP-GlcNAc [uridine 5′-(2-acetamido-2-deoxy-D-glucopyranosyl diphosphate)] acts as the donor of the GlcNAc residue while a hydroxy group situated at a specific site of the growing oligosaccharide chain serves as the acceptor [9].

**Figure 1:**
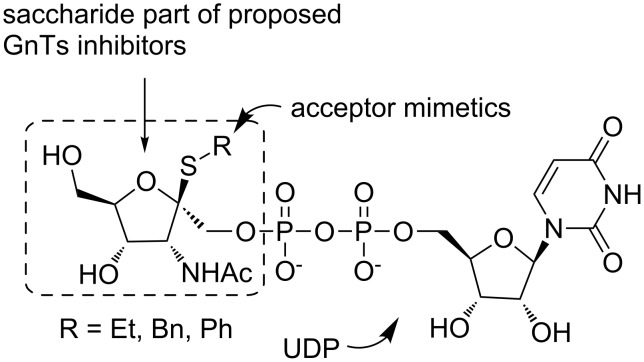
Proposed structure of GnTs inhibitors.

The target-directed search for effective GnTs inhibitors based on the rational design of model compounds remains a difficult task due to the complexity of the GnTs catalytic mechanism. The main reasons originating from the complex character of the catalytic mechanism which complicate the search for GnTs inhibitors are a) participation of four components in the transition state (sugar donor, acceptor, nucleotide and metal co-factor), b) weak binding of the enzymes to natural substrates, and c) lack of structural data [10]. Various methods have been applied in order to unveil effective GTs inhibitors [11–16]. The most important approaches are based on the design of acceptor analogues, donor analogues and transition state mimetics. Despite all the effort and the numerous GTs inhibitors identified, only a few achieved significant activity. On the basis of the previous investigations, a new class of GTs inhibitors imitating carbohydrates has been revealed [17]. These structurally modified carbohydrates are designed to simulate the shape and functionality of the natural substrates in the ground and/or the transition state (TS) of the enzymatic reaction [14]. In the case of GTs inhibitors, the carbohydrate mimetics that imitate the TS of the enzymatic reactions should exhibit a higher inhibition activity than the natural carbohydrate substrates [9,16]. Investigations of the catalytic mechanism of inverting glycosyltransferases [18] and *N*-acetylglucosaminyltransferase I [19] by employing ab initio calculations resulted in the design of inhibitors based on carbohydrate mimetics that simulate the transition state of the enzymatic reaction (Figure 1).

Within the structure of the proposed inhibitors of *N*-acetylglucosaminyltransferases (Figure 1), the ethyl, benzyl and/or phenyl groups imitate the acceptor substrate and the “1-thio” linker provides the proper distance between the “donor” and the “acceptor” together with the charge distribution as it was calculated for the TS [9–10].

In this contribution, attention is focused on the synthesis of the saccharide moiety of potential GnTs inhibitors (framed structure in Figure 1). In this respect, a novel approach to 3-acetamido-3-deoxy-D-psicofuranose derivatives, based on the transformation of D-mannose, is described. In addition, the thioglycosylation of fully protected 3-acetamido-3-deoxy-D-psicofuranose **11** with ethanethiol was examined under various conditions.

## Results and Discussion

**Scheme 1:**
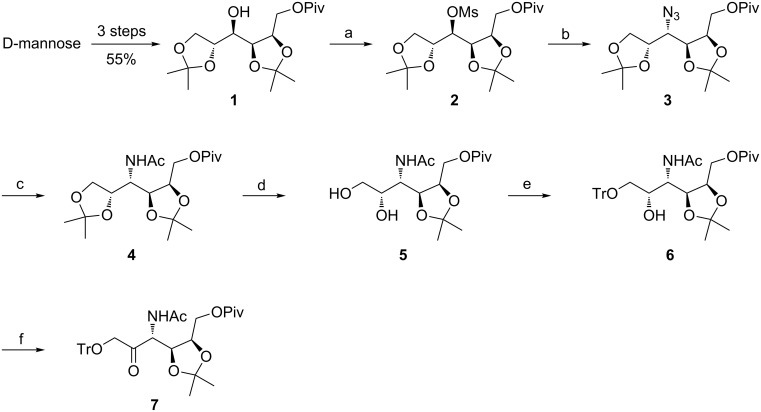
Reagents and conditions: a) MsCl, Et_3_N, CH_2_Cl_2_, 0 °C → rt, overnight, 93%; b) NaN_3_, H_2_O/DMF 1:20 (v/v), reflux, 9 h, 23%; c) i: Zn/NH_4_Cl, H_2_O/EtOH 1:3 (v/v), rt, 2 h, ii: AcCl, pyridine, CH_2_Cl_2_, rt, 30 min, 80% (2 steps); d) 70% AcOH, rt, overnight, 71%; e) TrCl, Et_3_N, CH_2_Cl_2_, 0 °C → rt, overnight, 88%; f) DMP, CH_2_Cl_2_, rt, overnight, 95%.

The synthesis of the saccharide moiety of potential GnTs inhibitors started from commercially available D-mannose. The latter was transformed into suitably protected 1,2:4,5-di-*O*-isopropylidene-6-*O*-pivaloyl-D-mannitol (**1**) in three steps by the procedures described in the literature [20–22]. The standard preparation of mesylate with methanesulfonyl chloride and Et_3_N as base smoothly provided 1,2:4,5-di-*O*-isopropylidene-3-*O*-methanesulfonyl-6-*O*-pivaloyl-D-mannitol (**2**) in 93% yield (Scheme 1).

**Scheme 2:**
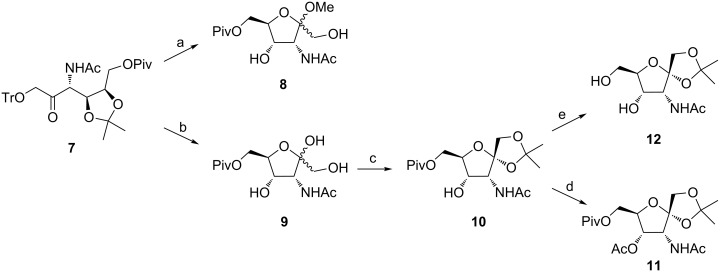
Reagents and conditions: a) H_2_SO_4_, MeOH, rt, overnight, 71%; b) H_2_SO_4_, 70% AcOH, 0 °C → rt, 4 h, 84%; c) 2,2-dimethoxypropane, acetone, PTSA, rt, 4 h, 63%; d) Ac_2_O, pyridine, CH_2_Cl_2_, rt, overnight, 92%; e) MeONa, MeOH, rt, overnight, 97%.

In the next step, the treatment of mesylate **2** with sodium azide in refluxing aqueous DMF afforded 3-azido-3-deoxy-1,2:4,5-di-*O*-isopropylidene-6-*O*-pivaloyl-D-altritol (**3**) in a low yield (23%). The addition of a catalytic amount of tetrabutylammonium chloride (0.15 equiv) to the reaction mixture in order to form tetrabutylammonium azide in situ did not improve the yields of the substitution. In the course of the nucleophilic substitution, unidentified elimination products were observed as the prevailing constituents of the reaction mixture together with the desired 3-azido derivative **3**. The majority of the byproducts were separated by column chromatography on silica gel (EtOAc/hexane 1:10). A complete separation of the elimination products from **3** was not necessary since they were readily removed in the next step. An analytically pure sample of **3** was obtained by additional column chromatography. In order to increase the yield of azide **3** by replacing 3-*O*-mesylate **2** with the corresponding 3-*O*-triflate in the nucleophilic substitution reaction with sodium azide, the *O*-protected mannitol derivative **1** was treated with triflic anhydride in the presence of pyridine. However, the resulting 3-*O*-triflate was unstable and decomposed rapidly after the work-up. On the other hand, an attempted tosylation of alcohol **1** using TsCl and pyridine in CH_2_Cl_2_ did not proceed, probably due to steric hindrance, and only the starting material was recovered. Next, the azide **3** (contaminated with some elimination products) was subjected to reduction with powdered Zn and NH_4_Cl as reducing agents [23] followed by acetylation to afford 3-acetamido-3-deoxy-1,2:4,5-di-*O*-isopropylidene-6-*O*-pivaloyl-D-altritol (**4**) in a good yield. It should be mentioned that the reduction of compound **3** under the Staudinger protocol (PPh_3_, H_2_O/THF) was found to be slow and ineffective. The following selective acidic hydrolysis of 1,2-*O*-isopropylidene ketal in **4** using 70% AcOH led to 3-acetamido-3-deoxy-4,5-*O*-isopropylidene-6-*O*-pivaloyl-D-altritol (**5**). Subsequently, the selective protection of the primary hydroxy group in diol **5** as trityl ether under standard conditions followed by the oxidation of the free secondary hydroxy group in 3-acetamido-3-deoxy-4,5-*O*-isopropylidene-6-*O*-pivaloyl-1-*O*-trityl-D-altritol (**6**) with Dess–Martin periodinane yielded a protected open-chain form of 3-acetamido-3-deoxy-D-psicose, namely 3-acetamido-3-deoxy-4,5-*O*-isopropylidene-6-*O*-pivaloyl-1-*O*-trityl-D-psicose (**7**) in an excellent yield (Scheme 1). This compound represents the key intermediate for the further synthesis of 3-acetamido-3-deoxy-D-psicofuranose derivatives bearing variously protected or unprotected hydroxy groups on the furanose ring. For example, the treatment of **7** with concentrated H_2_SO_4_ in methanol resulted in a mixture of anomeric methyl 3-acetamido-3-deoxy-6-*O*-pivaloyl-α/β-D-psicofuranosides (**8**) in 71% yield (Scheme 2).

**Figure 2:**
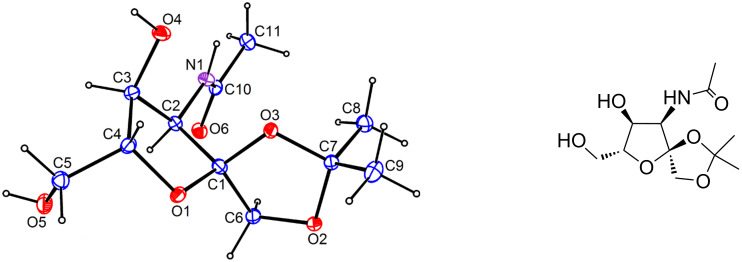
Molecular structure (ORTEP drawing with adjacent ChemDraw image) of compound **12**. Atomic displacement ellipsoids are drawn at 30% probability level.

Based on the integration of the ^1^H NMR signals of the acetamide group in derivatives **8**, a 3:2 ratio of the anomers was determined, albeit without the exact assignment of anomeric configurations to particular anomers. In the context of the synthesis of the saccharide part of GnTs inhibitors, psicose derivative **7** was subjected to a simultaneous hydrolysis of the trityl ether and the 4,5-*O*-isopropylidene ketal with concentrated H_2_SO_4_ in 70% AcOH to afford 3-acetamido-3-deoxy-6-*O*-pivaloyl-α/β-D-psicofuranose (**9**) in 84% yield. In the next step, the hydroxy groups of **9** in positions 1 and 2 were protected as isopropylidene ketal. Although both anomers were formed together with 3-acetamido-3-deoxy-4,5-*O*-isopropylidene-6-*O*-pivaloyl-D-psicose as a byproduct originating from isopropylidenation of the open-chain form of **9**, only the α-anomer, namely 3-acetamido-3-deoxy-1,2-*O*-isopropylidene-6-*O*-pivaloyl-α-D-psicofuranose (**10**), was isolated in 63% yield. The β-anomer was detected in a negligible amount and could not be separated from the above-mentioned byproduct. Acetylation of the 4-hydroxy group in derivative **10** under standard conditions yielded fully protected 3-acetamido-4-*O*-acetyl-3-deoxy-1,2-*O*-isopropylidene-6-*O*-pivaloyl-α-D-psicofuranose (**11**) as the suitable substrate for thioglycosylation. Attempts to determine the configuration at the anomeric center in compounds **10** or **11** by employing NOE NMR technique were ambiguous. Although both derivatives **10** and **11** were crystalline solids, it was not possible to obtain suitable crystals for single-crystal X-ray analysis. However, removal of the pivaloyl group from derivative **10** afforded 3-acetamido-3-deoxy-1,2-*O*-isopropylidene-α-D-psicofuranose (**12**) which, after crystallization (EtOAc/hexane 1:2), afforded crystals suitable for X-ray analysis. This unambiguously confirmed the α-configuration at C-2 of furanose **12** (Figure 2). Consequently, based on this information, the α-configurations at the anomeric center of derivative **10** as well as **11** were determined.

**Scheme 3:**
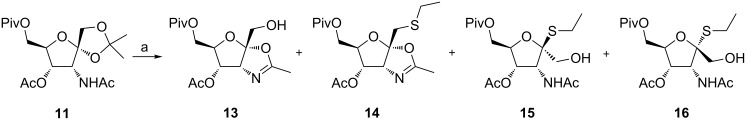
Reagents and conditions: a) EtSH, BF_3_·OEt_2_, CH_2_Cl_2_, −5 °C → rt, 4 h, **13**: 55%, **14**: 34%.

The crucial step leading to the target saccharide part of potential GnTs inhibitors represents the thioglycosylation of the fully protected psicofuranose derivative **11** with ethanethiol as the model thiolating agent. However, the treatment of **11** with EtSH and BF_3_·OEt_2_ led predominantly to 2-methyloxazoline derivatives **13** and **14**, while the required ethyl 3-acetamido-3-deoxy-6-*O*-pivaloyl-2-thio-β-D-psicofuranoside (**15**) was detected only in traces as a mixture with the corresponding α-anomer **16** (Scheme 3).

Further attempts to achieve the thioglycosylation of D-psicofuranose derivative **11** under various conditions, including EtSH/TMSOTf/CH_2_Cl_2_, EtSH/TMSOTf and EtSH/CSA/CH_2_Cl_2_ did not improve the yield of the required 2-thio-β-D-psicofuranoside derivative **15**.

### X-ray analysis

Single crystals (stable at ambient temperature) that were suitable for X-ray diffraction measurements were obtained by slow crystallization of **12** from EtOAc/hexane 1:2 with cooling in a refrigerator. The preliminary orientation matrices and final cell parameters were obtained using the Siemens SMART and Siemens SAINT software [24]. The data were empirically corrected for absorption and other effects using the SADABS program [25]. The crystal and experimental data for compound **12** are summarized in Table 1. The structure was resolved by direct methods and refined by full-matrix least-squares on all *F*^2^ data using SHELXS-97 [26] and SHELXL2013 [27]. The non-H atoms were refined anisotropically.

**Table 1:** Crystallographic and experimental data^a^ for compound **12**.

Crystal data	

Empirical formula	C_11_H_19_NO_6_
Formula weight/mass	261.27
Crystal size	0.420 × 0.060 × 0.040 mm
Crystal description	needle
Crystal colour	colourless
Crystal system	monoclinic
Space group	*C*2
Unit cell dimensions	*a* = 18.8306(8) Å
	*b* = 5.4573(2) Å
	*c* = 13.1363(6) Å
Volume	1234.02(9) Å^3^
*Z*	4
Calculated density	1.406 Mg/m^3^
Absorption coefficient	0.114 mm^−1^
F(000)	560

Data collection	

Measurement device type	Bruker APEX-II CCD
Measurement method	ω-scans
Temperature	100(2) K
Wavelength	0.71073 Å
Monochromator	Graphite
θ range for data collection	1.696 to 27.102°
Index ranges	−24 <= *h* <= 24, −6 <= *k* <= 6, −16 <= *l* <= 16
Reflections collected/unique	24125/2712 [*R*(int) = 0.0592]
Completeness to θ = 25.000°	100.0%
Absorption correction	Semi-empirical from equivalents
Max. and min. transmission	0.9705 and 0.9123

Refinement	

Refinement method	Full-matrix least-squares on *F*^2^
Data/restraints/parameters	2712/3/179
Goodness-of-fit on *F*^2^	1.065
Final *R* indices [*I*>2σ(I)]	*R*_1_ = 0.0338, *wR*_2_ = 0.0664
*R* indices (all data)	*R*_1_ = 0.0418, *wR*_2_ = 0.0698
Largest diff. peak and hole	0.163 and −0.176 *e*·Å^−3^

^a^Standard deviations are given in parentheses.

Based on the calculated values of the ring-puckering parameters [28] *Q* = 0.376(2) Å and Φ = 50.6(4)° and relevant torsion angles (Table 2), the five-membered O1–C1–C2–C3–C4 furanose ring (O5–C2–C3–C4-C5 according to the IUPAC hexofuranose nomenclature [29]) in **12** adopts the ^2^*T*_1_ conformation (^C2^*T*_C1_, twisted on C1–C2, C1-*exo*, C2-*endo*). For the five-membered 1,3-dioxolane ring (O2–C6–C1–O3–C7), the puckering parameters *Q* = 0.288(2) Å and Φ = 15.7(5)° and relevant torsion angles (Table 2) are indicative of ^O^*T*_1_ conformation (^O2^*T*_C6_, twisted on O2–C6, C6-*exo*, O2-*endo*). The absolute configuration at chiral atoms C2, C3, and C4 of the O1–C1–C2–C3–C4 furanose ring (Figure 2) was assigned on the basis of the known arrangement in analogous D-psicofuranose derivatives. The α-D-configuration at the anomeric atom C1 was established on the basis of inspection of the relevant torsion angles (Table 2).

**Table 2:** Relevant torsion angles (°)^a^ for the five-membered furanose ring, five-membered 1,3-dioxolane ring and anomeric center in compound **12**.

Ring	Torsion angle	Value

Furanose	O1–C1–C2–C3	39.5(2)
	C1–C2–C3–C4	−28.8(2)
	C2–C3–C4–O1	9.0(2)
	C3–C4–O1–C1	16.6(2)
	C4–O1–C1–C2	−34.9(2)
1,3-Dioxolane	O2–C6–C1–O3	24.0(2)
	C6–C1–O3–C7	−7.9(2)
	C1–O3–C7–O2	−11.2(2)
	O3–C7–O2–C6	26.9(2)
	C7–O2–C6–C1	−31.6(2)
Anomeric center	C3–C2–C1–O3	−78.7(2)
	C3–C2–C1–C6	162.24(18)
	C4–O1–C1–O3	83.7(2)
	C4–O1–C1–C6	−161.35(19)
	N1–C2–C1–O3	44.1(2)
	N1–C2–C1–C6	−75.0(2)

^a^Standard deviations are given in parentheses.

## Conclusion

In summary, a new synthetic strategy leading to the formation of a 3-acetamido-3-deoxy-D-psicofuranose frame is presented. The attempted thioglycosylation of fully protected 3-acetamido-3-deoxy-α-D-psicofuranose **11** resulted in ring closure of the acetamido group, affording 2-methyloxazoline derivatives **13** and **14**. Further research into an alternative strategy for the synthesis of 3-azido-3-deoxy-D-psicofuranose derivatives will be performed so as to avoid the formation of the 2-methyloxazoline ring.

## Supporting Information

File 1 Experimental procedures and spectral data.
