# Supplementary material for: Towards inhibitors of glycosyltransferases: A novel approach to the synthesis of 3-acetamido-3-deoxy-D-psicofuranose derivatives
Source: Beilstein J Org Chem. 2015 Sep 4;11:1547–52. doi: 10.3762/bjoc.11.170 (PMC4578356; doi:10.3762/bjoc.11.170)
Supplement: File 1 — Experimental procedures and spectral data. [file Beilstein_J_Org_Chem-11-1547-s001.pdf]

Supporting Information

for

Towards inhibitors of glycosyltransferases: A novel approach to the synthesis of 3-acetamido-3-deoxy-D-psicofuranose derivatives

Maroš Bella*[1], Miroslav Koóš[1] and Chun-Hung Lin[2]

Address: [1]Institute of Chemistry, Slovak Academy of Sciences, Dúbravská cesta 9, SK-845 38, Bratislava, Slovakia and [2]Institute of Biological Chemistry, Academia Sinica, No. 128 Academia Road Sec. 2, Taipei 11529, Taiwan

Email: Maroš Bella - chemmajo@savba.sk

* Corresponding author

Experimental procedures and spectral data

Experimental

General: The starting D-mannitol derivative **1** was prepared from D-mannose according to published methods [1-3]. All reagents were commercially available and were used as received. All solvents were of technical grade and were distilled before use. Dichloromethane (Slavus, Slovakia) was boiled under reflux over P_2O_5 for 1 h and was distilled immediately prior to use. Melting points were determined using a Boetius PHMK 05 microscope. Specific optical rotations were determined on a Perkin Elmer 241 polarimeter. ^{1}H and ^{13}C NMR spectra were recorded on a VNMRS 400 MHz Varian spectrometer equipped with 5 mm ^{1}H–^{19}F/^{15}N–^{31}P PFG AutoX DB NB probe head operating at 400 MHz and 100 MHz working frequencies, respectively. Chemical shifts are given in ppm (δ) relative to residual signal of appropriate deuterated solvent used ($CDCl_3$, CD_3OD). Coupling constants are given in Hz. All given ^{13}C spectra are proton decoupled. The 2D NMR experiments (HSQC and COSY) were used for the signal assignments for compounds **9**–**14**. Letters a and b denote two non-equivalent protons in CH_2 groups. Microanalyses were performed on a Fisons EA 1108 analyser. The IR spectra (ATR) were measured using a Nicolet 6700 FTIR spectrometer. X-ray diffraction was performed on an X8 APEX Bruker diffractometer. Thin-layer chromatography (TLC) was performed on glass plates precoated with TLC Silica gel 60 F_{254} (E. Merck). Visualization was achieved by immersing the plates into 10% solution of phosphomolybdic acid (PMA) in ethanol and heating at ca 200 °C with a heat gun. Column chromatography was performed as flash chromatography on Silica gel 60 (E. Merck, 0.040–0.063 mm). Solvents used for flash chromatography were of technical grade and were distilled before use.

1,2:4,5-Di-*O*-isopropylidene-3-*O*-methanesulfonyl-6-*O*-pivaloyl-D-mannitol (2): Et_3N (45 mL, 0.322 mol) was added to a stirred solution of D-mannitol **1** (74.3 g, 0.214 mol) in dry CH_2Cl_2 (600 mL) and the resulting solution was cooled in an ice bath to 0 °C. Methanesulfonyl chloride (23.2 mL, 0.30 mol) was then added dropwise while cooling in the ice bath. Once the methanesulfonyl chloride was added, the ice bath was removed and the stirring was continued at room temperature overnight. Once the reaction was complete, the reaction mixture was washed with water (3 × 500 mL), the organic layer was separated, dried with Na_2SO_4, filtered and the solvent was evaporated under reduced pressure. The crude oily product was purified by column chromatography on silica gel using EtOAc/hexane 1:4 → 2:1 as an eluent to afford mesylate **2** (85.0 g, 93%) as a yellowish oil; R_f = 0.27 (EtOAc/hexane 1:4); $[\alpha]_D^{20}$ = +19.4 (*c* 1, MeOH), ^{1}H NMR (400 MHz, $CDCl_3$): δ 4.83 (m, 1H), 4.41–4.12 (m, 6H), 4.08–3.97 (m, 1H), 3.13 (s, 3H, OSO_2CH_3), 1.48, 1.42, 1.36 and 1.35 [4s, each 3H, $C(CH_3)_2$], 1.22 [s, 9H, $C(CH_3)_3$] ppm; ^{13}C NMR (100 MHz, $CDCl_3$): δ 178.1 (C=O), 110.3 and 109.1 (CMe_2), 79.0 (C-3), 76.5, 74.9, 74.6, 66.7, 62.7, 39.3 (OSO_2CH_3), 38.7 [$C(CH_3)_3$], 27.1, 25.9, 25.6 and 25.2 [$C(CH_3)_2$] ppm; IR (ATR) $\tilde{\nu}$: 2982, 1727 (C=O), 1355, 1170, 1150, 944, 514 cm^{-1}; anal. calcd (%) for $C_{18}H_{32}O_9S$: C, 50.93; H, 7.60; S, 7.55; found (%): C, 50.84; H, 7.63; S 7.52.

3-Azido-3-deoxy-1,2:4,5-di-*O*-isopropylidene-6-*O*-pivaloyl-D-altritol (3): To a solution of mesylate **2** (85.0 g, 0.20 mol) in DMF (1000 mL), water (50 mL) was added, followed by addition of NaN_3 (65.0 g, 1.0 mol) and the resulting mixture was heated under reflux for 9 h. The solvent was then evaporated under reduced pressure and the residue was dissolved in EtOAc (600 mL) and water (600 mL). The organic layer was separated, washed with water (3 × 600 mL), dried over Na_2SO_4, filtered and the solvent was evaporated under reduced pressure. The crude oily

product was separated from elimination products by column chromatography on silica gel using EtOAc/hexane 1:10 as an eluent to afford azide **3** (17.1 g, 23%) as a colorless oil contaminated with some elimination products. An analytical sample of **3** was obtained by additional column chromatography; R_f = 0.53 (EtOAc/hexane 1:4); $[\alpha]_D^{20}$ = +23.0 (*c* 1, MeOH); ^{1}H NMR (400 MHz, $CDCl_3$): δ 4.43–4.31 (m, 3H), 4.22 (dd, *J* = 11.7, 5.4 Hz, 1H), 4.12–4.09 (m, 2H), 3.94 (dd, *J* = 8.9, 6.4 Hz, 1H), 3.30 (dd, *J* = 10.2, 6.1 Hz, 1H), 1.47, 1.43, 1.39 and 1.33 [4s, each 3H, $C(CH_3)_2$], 1.23 [s, 9H, $C(CH_3)_3$] ppm; ^{13}C NMR (100 MHz, $CDCl_3$): δ 178.1(C=O), 109.4 and 109.2 (CMe_2), 77.5, 75.2, 75.0, 66.9, 62.4, 60.9, 38.7 [$C(CH_3)_3$], 27.4, 27.1, 26.1 and 25.1 [$C(CH_3)_2$] ppm; IR (ATR) $\tilde{\nu}$: 2983, 2110 (N_3), 1729 (C=O), 1214, 1150, 1064, 854, 510 cm^{-1}; anal. calcd (%) for $C_{17}H_{29}N_3O_6$: C, 54.97; H, 7.87; N, 11.31; found (%): C, 55.09; H, 7.82; N, 11.26.

3-Acetamido-3-deoxy-1,2:4,5-di-*O*-isopropylidene-6-*O*-pivaloyl-D-altritol (4): To a solution of azide **3** (8.1 g, 21.8 mmol) in EtOH (70 mL), water (22 mL) was added followed by NH_4Cl (2.92 g, 54.5 mmol). Once the NH_4Cl was dissolved, Zn powder (3.56 g, 54.5 mmol) was added in one portion while stirring vigorously. The temperature of the reaction mixture raised spontaneously to 45–50 °C and stirring was continued for 2 h. The mixture was then filtered with suction and the filter cake was washed with EtOH (120 mL). The solvent was evaporated under reduced pressure and the residue was dissolved in EtOAc (200 mL). The organic layer was washed with water (200 mL), separated, dried over Na_2SO_4, filtered and the solvent was evaporated under reduced pressure. The oily residue was dissolved in CH_2Cl_2 (150 mL) and to the resulting solution, pyridine (1.8 mL, 22.3 mmol) was added followed by addition of AcCl (1.6 mL, 22.5 mmol). After 30 min of stirring, the mixture was treated with MeOH (2 mL) and washed with water (150 mL). The organic layer

was separated, dried over Na_2SO_4, filtered and the solvent was evaporated under reduced pressure. The residue was co-evaporated with toluene (3 × 50 mL) and the crude product was purified by column chromatography on silica gel using EtOAc/hexane 2:1 as an eluent to afford acetamido derivative **4** (6.75 g, 80%) as a white solid; mp 117–118 °C; R_f = 0.38 (EtOAc/hexane 2:1); $[\alpha]_D^{20}$ = + 56.4 (*c* 1, MeOH); ^{1}H NMR (400 MHz, $CDCl_3$): δ 5.61 (d, *J* = 8.9 Hz, 1H, NH), 4.53 (t, *J* = 6.9 Hz, 1H), 4.33 (dd, *J* = 11.5, 3.0 Hz, 1H), 4.28 (ddd, *J* = 8.1, 5.1, 3.1 Hz, 1H), 4.22−4.13 (m, 2H), 4.10 (dd, *J* = 11.5, 3.0 Hz, 1H), 4.01 (dd, *J* = 8.6, 7.1 Hz, 1H), 3.61 (dd, *J* = 8.6, 6.5 Hz, 1H), 2.04 (s, 3H, $COCH_3$), 1.47, 1.43, 1.36 and 1.35 [s, 3H, $C(CH_3)_2$], 1.21 [s, 9H, $C(CH_3)_3$] ppm; ^{13}C NMR (100 MHz, $CDCl_3$): δ 178.1 (C=O), 170.2 (NHC=O), 109.1 (2×*C*Me$_2$), 75.6, 75.4, 74.3, 65.8, 62.5, 48.2, 38.7 [$C(CH_3)_3$], 27.1 [$C(CH_3)_3$], 27.8, 26.3, 25.6 and 24.6 [$C(CH_3)_2$], 23.4 ($NHCOCH_3$) ppm; IR (ATR) $\tilde{\nu}$: 3254, 2976, 1729 (C=O), 1636, 1548, 1373, 1148, 1055, 855 cm^{-1}; anal. calcd (%) for $C_{19}H_{33}NO_7$: C, 58.90; H, 8.85; N, 3.61. Found (%): C, 58.81; H, 8.91; N, 3.66.

3-Acetamido-3-deoxy-4,5-*O*-isopropylidene-6-*O*-pivaloyl-D-altritol (5): A solution of acetamide **4** (6.75 g, 17.4 mmol) in 70% AcOH (120 mL) was stirred at room temperature overnight. The reaction mixture was diluted with EtOAc (200 mL) and carefully alkalized with aqueous Na_2CO_3 solution (150 g of Na_2CO_3 in 400 mL of water). The organic layer was separated and the aqueous layer was extracted with EtOAc (1 × 150 mL). The combined extracts were dried over Na_2SO_4, filtered and the solvent was evaporated under reduced pressure. The crude product was purified by column chromatography on silica gel using EtOAc/MeOH 20:1 as an eluent to afford diol **5** (4.33 g, 71%) as a colorless oil; R_f = 0.10 (EtOAc); $[\alpha]_D^{20}$ = +50.1 (*c* 1, MeOH); ^{1}H NMR (400 MHZ, $CDCl_3$): δ 6.28 (d, *J* = 9.6 Hz, 1H, NH), 4.38–4.25 (m, 3H), 4.21 (td, *J* = 9.6, 1.6 Hz, 1H), 4.14 (t, *J* = 6.6 Hz, 1H), 4.08 (dd, *J* = 11.8, 7.1 Hz, 1H), 3.54

(dd, J = 11.4, 6.7 Hz, 1H), 3.41 (dd, J = 11.4, 7.4 Hz, 1H), 3.09 (br s, 1H, OH), 2.05 (s, 3H, NHCOCH_3), 1.48 and 1.36 [2s, each 3H, C(CH_3)$_2$], 1.21 [s, 9H, C(CH_3)$_3$] ppm; ^{13}C NMR (100 MHz, CDCl$_3$): δ 178.4 (C=O), 171.5 (NHC=O), 109.1 (*C*Me$_2$), 75.4, 75.0, 70.1, 62.7, 62.5, 48.5, 38.7 [*C*(CH$_3$)$_3$], 27.1 [C(*C*H$_3$)$_3$], 27.6 and 25.4 [C(*C*H$_3$)$_2$], 23.0 (NHCO*C*H$_3$) ppm; IR (ATR) $\tilde{\nu}$: 3360, 2978, 1713 (C=O), 1642, 1544, 1173, 1039, 1029, 871 cm^{-1}; anal. calcd (%) for $C_{16}H_{29}NO_7$: C, 55.32; H, 8.41; N, 4.03; found (%): C, 55.40; H, 8.34; N, 4.10.

3-Acetamido-3-deoxy-4,5-*O*-isopropylidene-6-*O*-pivaloyl-1-*O*-trityl-D-altritol (6): To a stirred solution of diol **5** (4.3 g, 12.4 mmol) in dry CH_2Cl_2 (150 mL), Et_3N (2.6 mL, 18.6 mmol) was added and the resulting solution was cooled in an ice bath to 0 °C. Then trityl chloride (4.2 g, 15.0 mmol) was added in one portion while cooling in the ice bath. After stirring for 15 min the ice bath was removed and the stirring was continued at room temperature overnight. The reaction mixture was washed with water (2 ×150 mL). The separated organic layer was dried with Na_2SO_4, filtered and the solvent was evaporated under reduced pressure. The crude foamy product was purified by column chromatography on silica gel using EtOAc/hexane 1:1 as an eluent to afford trityl derivative **6** (6.5 g, 88%) as a white amorphous material; R_f = 0.20 (EtOAc/hexane 1:1); $[\alpha]_D^{20}$ = +14.9 (*c* 1, MeOH); ^{1}H NMR (400 MHz, CDCl$_3$): δ 7.44–7.36 (m, 6H, Ph), 7.34–7.19 (m, 9H, Ph), 5.79 (d, J = 9.4 Hz, 1H, NH), 4.33–4.07 (m, 6H), 3.20 (dd, J = 9.4, 5.6 Hz, 1H), 3.07 (dd, J = 9.4, 7.9 Hz, 1H), 2.88 (d, J = 3.1 Hz, 1H, OH), 1.80 (s, 3H, NHCOCH_3), 1.47 and 1.32 [2s, each 3H, C(CH_3)$_2$], 1.18 [s, 9H, C(CH_3)$_3$] ppm; ^{13}C NMR (100 MHz, CDCl$_3$): δ 178.0 (C=O), 169.7 (NHC=O), 143.4, 128.6, 127.9 and 127.1 (Ph), 108.9 (*C*Me$_2$), 87.1 (*C*Ph$_3$), 76.2, 75.3, 69.4, 64.1, 62.5, 48.7, 38.7 [*C*(CH$_3$)$_3$], 27.1 [C(*C*H$_3$)$_3$], 27.5 and 25.2 [C(*C*H$_3$)$_2$], 23.1 (NHCO*C*H$_3$) ppm; IR (ATR) $\tilde{\nu}$: 3351, 2977, 1722 (C=O), 1656, 1155, 1069, 700

cm^{-1}; anal. calcd (%) for $C_{35}H_{43}NO_7$: C, 71.28; H, 7.35; N, 2.38; found (%): C, 71.36; H, 7.40; N, 2.34.

3-Acetamido-3-deoxy-4,5-*O*-isopropylidene-6-*O*-pivaloyl-1-*O*-trityl-D-psicose (7): To a stirred solution of trityl derivative **6** (6.5 g, 11.0 mmol) in CH_2Cl_2 (200 mL), a 15% solution of Dess–Martin periodinane in CH_2Cl_2 (34 mL, 16.5 mmol) was added dropwise and stirring was continued overnight. Afterwards, a saturated $NaHCO_3$ solution (100 mL) and a 25% solution of $Na_2S_2O_3$ (100 mL) were added and the mixture was shaken in a separatory funnel. The organic layer was separated, dried over Na_2SO_4, filtered and the solvent was evaporated under reduced pressure. The crude foamy product was purified by column chromatography on silica gel using EtOAc/hexane 1:1 as an eluent to afford D-psicose derivative **7** (6.2 g, 95%) as a white amorphous material; R_f = 0.25 (EtOAc/hexane1:1); $[\alpha]_D^{20}$ = +39.2 (*c* 1, MeOH); ^{1}H NMR (400 MHz, $CDCl_3$): δ 7.49–7.39 (m, 6H, Ph), 7.34–7.19 (m, 9H, Ph), 6.17 (d, *J* = 9.1 Hz, 1H, NH), 4.76 (dd, 1H, *J* = 10.0, 9.1 Hz, H-3), 4.33–4.21 (m, 2H), 4.24 (d, *J* = 18.1 Hz, 1H, H-1a), 4.12 (dd, *J* = 9.8, 5.6 Hz, 1H), 4.08 (dd, *J* = 12.7, 7.4 Hz, 1H, H-6a), 3.93 (d, *J* = 18.1 Hz, 1H, H-1b), 1.94 (s, 3H, NHCOCH_3), 1.22 and 1.21 [2s, each 3H, C(CH_3)$_2$], 1.17 [s, 9H, C(CH_3)$_3$] ppm; ^{13}C NMR (100 MHz, $CDCl_3$): δ 204.7 (C=O), 178.0 (C=O), 169.5 (NHC=O), 143.1, 128.5, 127.9 and 127.1 (Ph), 109.3 (*C*Me$_2$), 87.2 (*C*Ph$_3$), 76.4, 75.3, 70.8 (C-1), 62.2 (C-6), 52.5 (C-3), 38.6 [*C*(CH$_3$)$_3$], 27.1 [C(*C*H$_3$)$_3$], 25.0 [2×C(*C*H$_3$)$_2$], 23.0 (NHCO*C*H$_3$) ppm; IR (ATR) $\tilde{\nu}$: 3266, 2977, 1729 (C=O), 1681, 1655, 1151, 1081, 700 cm^{-1}; anal. calcd (%) for $C_{35}H_{41}NO_7$: C, 71.53; H, 7.03; N, 2.38; found (%): C, 71.60; H, 7.09; N, 2.42.

Methyl 3-acetamido-3-deoxy-6-*O*-pivaloyl-α/β-D-psicofuranoside (8): To a stirred solution of D-psicose derivative **7** (1.5 g, 2.55 mmol) in MeOH (35 mL), concentrated

H_2SO_4 (0.5 mL) was added dropwise and stirring was continued at room temperature overnight. After the reaction was completed, saturated Na_2CO_3 solution (5 mL) was added and the mixture was stirred for 20 min. The resulting suspension was filtered with suction and the filter cake was washed with MeOH (20 mL). Silica gel (8 g) was added to the filtrate and the solvent was evaporated under reduced pressure. The residue was loaded on a column of silica gel and eluted with a mixture of $CHCl_3$/MeOH 20:1 to afford an anomeric mixture (in the ratio of 3:2) of methyl furanosides **8** (0.58 g, 71%) as a colorless oil; R_f = 0.22 ($CHCl_3$/MeOH 20:1); ^1H NMR (400 MHz, CD_3OD): δ 4.46–4.37 (m, 3H), 4.23–4.19 (m, 4H), 4.15–4.10 (m, 3H), 3.66–3.49 (m, 4H), 3.32 and 3.29 (2s, each 3H, OCH_3), 2.06 and 2.03 (2s, each 3H, $NHCOCH_3$), 1.22 [s, 18H, $C(CH_3)_3$] ppm; ^{13}C NMR (100 MHz, CD_3OD): δ 179.79 and 179.75 (2×C=O), 174.6 and 173.4 (2×NHC=O), 110.2 and 107.1 (2×C-2), 84.7, 82.7, 71.82, 71.68, 66.3, 65.2, 61.3, 59.7, 58.4, 56.3, 49.19 and 49.05 (2×OCH_3), 39.88 and 39.86 [2×$C(CH_3)_3$], 27.59 and 27.54 [2×$C(CH_3)_3$], 22.59 and 22.57 (2×$NHCOCH_3$) ppm; IR (ATR) $\tilde{\nu}$: 3338, 2961, 1726 (C=O), 1648, 1526, 1155, 1032, 939 cm^{-1}; anal. calcd (%) for $C_{14}H_{25}NO_7$: C, 52.65; H, 7.89; N, 4.39; found (%): C, 52.70; H, 7.84; N, 4.42.

3-Acetamido-3-deoxy-6-*O*-pivaloyl-α/β-D-psicofuranose (9): A solution of D-psicose derivative **7** (1.0 g, 1.7 mmol) in 70% AcOH (25 mL) was cooled in an ice bath to 0 °C. Subsequently, concentrated H_2SO_4 (0.6 mL) was added dropwise under vigorous stirring and the mixture was stirred for 10 min. The ice bath was removed and stirring was continued at room temperature for 4 h. H_2SO_4 was then carefully neutralized with Na_2CO_3 (0.9 g) and the volatiles were evaporated under reduced pressure. The residue was co-evaporated with toluene (2 ×60 mL), MeOH (60 mL) was then added and the resulting suspension was treated with Na_2CO_3 (1.0 g). After

10 min of stirring, the mixture was filtered and the filter cake was washed with MeOH (10 mL). Silica gel (8 g) was added to the filtrate and the solvent was evaporated under reduced pressure. The residue was loaded on a column of silica gel and eluted with a mixture of $CHCl_3$/MeOH 10:1 to afford 1,2-diol **9** (0.44 g, 84%) as a colorless oil; R_f = 0.14 ($CHCl_3$/MeOH 10:1); $[\alpha]_D^{20}$ = –7.8 (*c* 1, MeOH); ^{1}H NMR (400 MHz, CD_3OD): δ 4.43 (d, *J* = 6.6 Hz, 1H, H-3), 4.27 (dt, *J* = 4.4, 2.5 Hz, 1H, H-5), 4.21 (dd, *J* = 11.8, 4.4 Hz, 1H, H-6a), 4.15 (dd, *J* = 11.8, 4.3 Hz, 1H, H-6b), 4.13 (dd, *J* = 6.6, 2.5 Hz, 1H, H-4), 3.49 (d, *J* = 11.8 Hz, 1H, H-1a), 3.42 (d, *J* = 11.8 Hz, 1H, H-1b), 2.04 (s, 3H, NHCOCH_3), 1.22 [s, 9H, C(CH_3)$_3$] ppm; ^{13}C NMR (100 MHz, CD_3OD): δ 179.9 (C=O), 173.5 (NHC=O), 105.1 (C-2), 84.2 (C-5), 72.3 (C-4), 65.5 (C-6), 64.4 (C-1), 54.4 (C-3), 39.8 [*C*(CH_3)$_3$], 27.5 [C(*C*H_3)$_3$], 22.6 (NHCO*C*H_3) ppm; IR (ATR) $\tilde{\nu}$: 3330, 2967, 1720 (C=O), 1645, 1531, 1155, 1036, 542 cm^{-1}; anal. calcd (%) for $C_{13}H_{23}NO_7$: C, 51.14; H, 7.59; N, 4.59; found (%): C, 51.08; H, 7.54; N, 4.63.

3-Acetamido-3-deoxy-1,2-*O*-isopropylidene-6-*O*-pivaloyl-α-D-psicofuranose (10): To a stirred solution of 1,2-diol **9** (0.44 g, 1.44 mmol) in acetone (8 mL), 2,2-dimethoxypropane (1 mL, 8.64 mmol) was added followed by addition of toluene-4-sulfonic acid monohydrate (36 mg, 0.19 mmol). After 4 h of stirring at room temperature, the mixture was alkalized with a few drops of conc. NH_4OH and the volatiles were evaporated under reduced pressure. The crude product was purified by column chromatography on silica gel using $CHCl_3$/MeOH 40:1 as an eluent to afford 1,2-*O*-isopropylidene-D-psicofuranose derivative **10** (0.32 g, 63%) as a thick colorless oil which crystallized slowly in a refrigerator; mp 89–91 °C; R_f = 0.15 ($CHCl_3$/MeOH 40:1); $[\alpha]_D^{20}$ = +47.9 (*c* 1, MeOH); ^{1}H NMR (400 MHz, $CDCl_3$): δ 6.08 (d, *J* = 9.0 Hz, 1H, NH), 4.47 (dd, *J* = 9.0, 6.2 Hz, 1H, H-3), 4.26 (td, *J* = 3.7, 1.9 Hz, 1H, H-5), 4.20 (dd, *J* = 11.9, 3.7 Hz, 1H, H-6a), 4.13 (dd, *J* = 11.9, 4.1 Hz, 1H, H-6b),

4.11 (br s, 1H, H-4), 4.06 (d, *J* = 9.4 Hz, 1H, H-1a), 4.00 (d, 1H, *J* = 9.4 Hz, H-1b), 2.96 (d, *J* = 7.9 Hz, 1H, OH), 2.05 (s, 3H, NHCOCH_3), 1.51 and 1.38 [2s, each 3H, C(CH_3)$_2$], 1.21 (s, 9H, C(CH_3)$_3$) ppm; ^{13}C NMR (100 MHz, $CDCl_3$): δ 178.1 (C=O), 170.2 (NHC=O), 111.5, 110.1, 83.4 (C-5), 71.4 (C-4), 70.3 (C-1), 63.8 (C-6), 53.1 (C-3), 38.8 [*C*(CH_3)$_3$], 27.1 [C(*C*H_3)$_3$], 26.4 and 26.1 [C(*C*H_3)$_2$], 23.1 (NHCO*C*H_3) ppm; IR (ATR) $\tilde{\nu}$: 3505, 2988, 1702 (C=O), 1658, 1515, 1164, 1022, 890 cm^{-1}; anal. calcd (%) for $C_{16}H_{27}NO_7$: C, 55.64; H, 7.88; N, 4.06; found (%): C, 55.58; H, 7.91; N, 4.01.

3-Acetamido-4-*O*-acetyl-3-deoxy-1,2-*O*-isopropylidene-6-*O*-pivaloyl-α-D-psicofuranose (11): To a stirred solution of D-psicofuranose derivative **10** (0.32 g, 0.92 mmol) in CH_2Cl_2 (7 mL), pyridine (1.1 mL, 13.6 mmol) was added followed by addition of acetic anhydride (0.85 mL, 9.0 mmol) and stirring was continued at room temperature overnight. MeOH (2 mL) was then added and stirring was continued for 15 min. The volatiles were evaporated under reduced pressure and the residue was co-evaporated with toluene (2 × 40 mL). The crude product was purified by column chromatography on silica gel using $CHCl_3$/MeOH 60:1 as an eluent to afford 4-*O*-acetylated D-psicofuranose derivative **11** (0.33 g, 92%) as a white solid; mp 105–107 °C; R_f = 0.22 ($CHCl_3$/MeOH 60:1); $[\alpha]_D^{20}$ = +27.9 (*c* 1, MeOH); ^{1}H NMR (400 MHz, $CDCl_3$): δ 5.78 (d, *J* = 9.5 Hz, 1H, NH), 5.06 (dd, *J* = 7.1, 1.8 Hz, 1H, H-4), 4.71 (dd, *J* = 9.5, 7.1 Hz, 1H, H-3), 4.31–4.19 (m, 3H, H-5, 2×H-6), 4.07 (d, *J* = 9.3 Hz, 1H, H-1a), 3.96 (d, *J* = 9.3 Hz, 1H, H-1b), 2.13 (s, 3H, OCOCH_3), 2.04 (s, 3H, NHCOCH_3), 1.49 and 1.40 [2s, each 3H, C(CH_3)$_2$], 1.21 (s, 9H, C(CH_3)$_3$) ppm; ^{13}C NMR (100 MHz, $CDCl_3$): δ 177.8 (C=O), 170.1 (NHC=O), 169.6 (C=O), 111.6, 109.9, 81.5 (C-5), 72.4 (C-4), 70.6 (C-1), 63.8 (C-6), 51.4 (C-3), 38.8 [*C*(CH_3)$_3$], 27.1 [C(*C*H_3)$_3$], 26.3 and 26.1 [C(*C*H_3)$_2$], 23.0 (NHCO*C*H_3), 20.9 (OCO*C*H_3) ppm; IR (ATR) $\tilde{\nu}$: 3260,

2980, 1739 (C=O), 1652, 1543, 1222, 1155, 1073, 904 cm^{-1}; anal. calcd (%) for $C_{18}H_{29}NO_8$: C, 55.80; H, 7.54; N, 3.62; found (%): C, 55.73; H, 7.48; N, 3.66.

3-Acetamido-3-deoxy-1,2-*O*-isopropylidene-α-D-psicofuranose (12): To a stirred solution of D-psicofuranose derivative **10** (0.44 g, 1.27 mmol) in MeOH (8 mL), a solution of MeONa in MeOH prepared from sodium (22 mg, 0.9 mmol) and MeOH (5 mL) was added and stirring was continued overnight. Solid NH_4Cl (87 mg) was then added and after 10 min of stirring, the volatiles were evaporated under reduced pressure. The crude product was purified by column chromatography on silica gel using $CHCl_3$/MeOH 10:1 as an eluent to afford diol **12** (0.32 g, 97%) as a white solid; mp 137–138 °C [EtOAc/hexane (1:2)]; R_f = 0.25 (10:1 $CHCl_3$–MeOH); $[\alpha]_D^{20}$ = +65.0 (*c* 1, MeOH); ^{1}H NMR (400 MHz, $CDCl_3$): δ 6.16 (d, *J* = 9.1 Hz, 1H, NH), 4.35 (dd, *J* = 9.1, 6.4 Hz, 1H, H-3), 4.19 (ddd, *J* = 7.6, 6.4, 2.1 Hz, 1H, H-4), 4.13 (d, *J* = 9.5 Hz, 1H, H-1a), 4.12 (dt, *J* = 3.8, 2.1 Hz, 1H, H-5), 3.98 (d, *J* = 9.5 Hz, 1H, H-1b), 3.69 (dd, *J* = 6.1, 3.8 Hz, 2H, 2×H-6), 3.45 (d, *J* = 7.6 Hz, 1H, OH-4), 3.15 (t, *J* = 6.1 Hz, 1H, OH-6), 2.03 (s, 3H, NHCOCH_3), 1.49 and 1.36 [2s, each 3H, C(CH_3)$_2$] ppm; ^{13}C NMR (100 MHz, $CDCl_3$): δ 170.7 (NHC=O), 111.4, 110.2, 86.1 (C-5), 70.8 (C-4), 70.4 (C-1), 62.5 (C-6), 53.4 (C-3), 26.5 and 26.1 [C(CH_3)$_2$], 23.1 (NHCOCH_3) ppm; IR (ATR) $\tilde{\nu}$: 3309, 2930, 1630, 1522, 1046, 873, 503 cm^{-1}; anal. calcd (%) for $C_{11}H_{19}NO_6$: C, 50.57; H, 7.33; N, 5.36; found (%): C, 50.62; H, 7.30; N, 5.40.

2-Methyl-4,5-dihydro-(4-*O*-acetyl-2,3-dideoxy-6-*O*-pivaloyl-α-D-psicofuranoso)[3,2-*d*]-1,3-oxazole (13) and 2-methyl-4,5-dihydro-(4-*O*-acetyl-1,2,3-trideoxy-1-ethylthio-6-*O*-pivaloyl-α-D-psicofuranoso)[3,2-*d*]-1,3-oxazole (14): To a stirred solution of D-psicofuranose derivative **11** (206 mg, 0.52 mmol) in dry CH_2Cl_2 (8 mL), EtSH (0.50 mL, 6.92 mmol) was added and the resulting solution

was cooled to −5 °C in an ice bath. $BF_3·OEt_2$ (0.18 mL, 1.46 mmol) was then added dropwise and after 15 min of stirring the ice bath was removed. The reaction mixture was stirred at room temperature for additional 4 h followed by the dilution with $CHCl_3$ (30 mL) and washing with saturated $NaHCO_3$ solution (40 mL). The organic layer was separated and the aqueous layer was extracted with additional portion of $CHCl_3$ (1 × 10 mL). The combined extracts were dried over Na_2SO_4, filtered and the solvent was evaporated under reduced pressure. The particular products were separated by column chromatography on silica gel using EtOAc/hexane 2:1→EtOAc as an eluent to afford oxazolines **13** (96 mg, 55%) and **14** (68 mg, 34%).

13: white solid; mp 151–152 °C [EtOAc/hexane (1:1)];; R_f = 0.10 (EtOAc); $[\alpha]_D^{20}$ = +104.3 (*c* 1, MeOH); ^{1}H NMR (400 MHz, $CDCl_3$): δ 4.95 (dd, *J* = 9.3, 7.0 Hz, 1H, H-4), 4.70 (d, *J* = 7.0 Hz, 1H, H-3), 4.30 (dd, *J* = 12.4, 2.8 Hz, 1H, H-6a), 4.19 (dd, *J* = 12.4, 4.6 Hz, 1H, H-6b), 4.09 (ddd, *J* = 9.3, 4.6, 2.8 Hz, 1H, H-5), 3.82–3.69 (m, 2H, 2×H-1), 2.87 (br t, *J* = 6.8 Hz, 1H, OH), 2.13 (s, 3H, $OCOCH_3$), 2.06 (d, *J* = 1.5 Hz, 3H, CH_3), 1.20 [s, 9H, $C(CH_3)_3$] ppm; ^{13}C NMR (100 MHz, $CDCl_3$): δ 178.1 (C=O), 170.0 (NHC=O), 166.6 (N=C), 115.7 (C-2), 77.3 (C-5), 72.8 (C-4), 70.2 (C-3), 62.5 (C-1), 61.9 (C-6), 38.8 [$C(CH_3)_3$], 27.0 [$C(CH_3)_3$], 20.6 ($OCOCH_3$), 14.2 (CH_3); IR (ATR) $\tilde{\nu}$: 3157, 2974, 1745, 1724 (C=O), 1667, 1234, 1137, 1082, 1037 cm^{-1}; anal.Calcd (%) for $C_{15}H_{23}NO_7$: C, 54.70; H, 7.04; N, 4.25; found (%): C, 54.77; H, 7.10; N, 4.20.

14: colorless oil; R_f = 0.15 (EtOAc/hexane 2:1); $[\alpha]_D^{20}$ = −24.5 (*c* 1, MeOH); ^{1}H NMR (400 MHz, $CDCl_3$): δ 4.98 (dd, *J* = 9.4, 7.0 Hz, 1H, H-4), 4.64 (dq, *J* = 7.0, 1.5 Hz, 1H, H-3), 4.30 (dd, *J* = 12.4, 3.0 Hz, 1H, H-6a), 4.20 (dd, *J* = 12.4, 4.7 Hz, 1H, H-6b), 4.05 (ddd, *J* = 9.4, 4.7, 3.0 Hz, 1H, H-5), 3.04 (d, *J* = 14.5 Hz, 1H, H-1a), 2.85 (d, *J* = 14.5 Hz, 1H, H-1b), 2.65 (q, *J* = 7.4 Hz, 2H, SCH_2CH_3), 2.14 (s, 3H, $OCOCH_3$), 2.06 (d, *J* = 1.5 Hz, 3H, CH_3), 1.25 (t, *J* = 7.4 Hz, 3H, SCH_2CH_3), 1.22 [s, 9H, $C(CH_3)_3$]

ppm; ^{13}C NMR (100 MHz, $CDCl_3$): δ 178.0 (C=O), 169.9 (NHC=O), 166.2 (N=C), 117.2 (C-2), 77.1 (C-5), 73.1 (C-4), 71.3 (C-3), 62.0 (C-3), 38.8 [$\mathit{C}(CH_3)_3$], 36.1 (C-1), 27.6 ($S\mathit{C}H_2CH_3$), 27.1 [$C(\mathit{C}H_3)_3$], 20.6 ($OCO\mathit{C}H_3$), 14.6 ($SCH_2\mathit{C}H_3$), 14.3 (CH_3); IR (ATR) $\tilde{\nu}$: 3361, 2968, 1729 (C=O), 1655, 1519, 1229, 1151, 1069, 1016 cm^{-1}; anal. calcd (%) for $C_{17}H_{27}NO_6S$: C, 54.67; H, 7.29; N, 3.75; S, 8.59; found (%): C, 54.74; H, 7.33; N, 3.69; S, 8.65.

References

1. Schmidt, O. T. *Methods Carbohyd*. *Chem*. **1963**, *2*, 318–324.

2. Shen, X.; Wu, Y.-L.; Wu, Y. *Helv*. *Chim*. *Acta* **2000**, *83*, 943–953.

3. Frick, W.; Krülle, T.; Schmidt, R. R. *Liebigs Ann*. *Chem*. **1991**, 435–438.
